# Treat the “Untreatable” by a Photothermal Agent: Triggering Heat and Immunological Responses for Rabies Virus Inactivation

**DOI:** 10.1002/advs.202205461

**Published:** 2022-11-17

**Authors:** Yujie Bai, Pei Huang, Na Feng, Yuanyuan Li, Jingbo Huang, Hongli Jin, Mengyao Zhang, Jingxuan Sun, Nan Li, Haili Zhang, Xianzhu Xia, Ben Zhong Tang, Hualei Wang

**Affiliations:** ^1^ Key Laboratory of Zoonosis Research Ministry of Education College of Veterinary Medicine Jilin University Changchun 130062 China; ^2^ Changchun Veterinary Research Institute Chinese Academy of Agricultural Sciences Changchun 130122 China; ^3^ School of Science and Engineering Shenzhen Institute of Aggregate Science and Technology The Chinese University of Hong Kong Shenzhen Guangdong 518172 China

**Keywords:** antivirus, immunological effects, organic photothermal agent, photothermal therapy, rabies virus

## Abstract

Rabies is a fatal neurological zoonotic disease caused by the rabies virus (RABV), and the approved post‐exposure prophylaxis (PEP) procedure remains unavailable in areas with inadequate medical systems. Although strategies have been proposed for PEP and postinfection treatment (PIT), because of the complexity of the treatment procedures and the limited curative outcome, developing an effective treatment strategy remains a holy grail in rabies research. Herein, a facile approach is proposed involving photothermal therapy (PTT) and photothermally triggered immunological effects to realize effective PEP and PIT simultaneously. The designed photothermal agent (N^+^TT‐*m*CB nanoparticles) featured positively charged functional groups and high photo‐to‐heat efficiency, which are favorable for virus targeting and inactivation. The level of the virus at the site of infection in mice is significantly decreased upon treatment with orthotopic PTT, and the transfer of the virus to the brain is significantly inhibited. Furthermore, the survival ratio of the mice three days postinfection is increased by intracranial injection of N^+^TT‐*m*CB and laser irradiation. Overall, this work provides a platform for the effective treatment of RABV and opens a new avenue for future antiviral studies.

## Graphical abstract

A facile approach that treats the untreatable rabies virus based on a photothermal agent is designed involving high photo‐to‐heat efficiency and positively charged functional groups. The virus is inactivated by photothermal therapy and photothermally triggered immunological effects which can realize effective post‐exposure prophylaxis and postinfection treatment simultaneously.

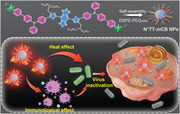

## 1 Introduction

Rabies is a fatal neurotropic zoonosis caused by rabies virus (RABV), which is in the genus *Lyssavirus*, family *Rhabdoviridae*. RABV enters the peripheral nerve at a bite site and is transported to the neurons of the central nervous system (CNS), resulting in severe neuroinflammation and 59 000 deaths annually.^[^ ^1^ ^]^ As there is currently almost no active therapy strategies against rabies, the clinical stages of rabies encephalitis remain untreatable.^[^ ^2^ ^]^ Although post‐exposure prophylaxis (PEP) can successfully prevent infection, it is still unavailable in some developing countries.^[^ ^3^ ^]^ The person who was bit always undergoes simple wound flushing for RABV disinfection, but only viruses on the surface can be inactivated. Viruses inside the bite site remain alive, resulting in considerable potential lethality to humans. Additional novel strategies, such as monoclonal antibody‐based approaches, aptamers and small interfering RNAs, have been proposed as forms of postinfection treatment (PIT) to combat rabies.^[^ ^4^ ^]^ However, a dominant hindrance to the treatment of rabies is its neurotropic characteristic, with the size exclusion limit of the blood–brain barrier (BBB) rendering the delivery of antiviral molecules and drugs to the CNS inherently problematic.^[^ ^5^ ^]^ Therefore, developing methods to inactivate internalized viruses by simple treatment and improve PIT remain the holy grail in rabies research.

Photothermal therapy (PTT) utilizes photothermal agents (PTAs) to absorb light energy and convert it into heat, and this method has been widely used in tumor treatment, antibacterial and antivirus research due to its advantages of deep penetration, significant efficacy, low toxicity, and few side effects.^[^ ^6^ ^]^ Local heat caused by photothermal effects exerts a biocidal effect by destroying the membrane of pathogens, leading to an increase in its permeability.^[^ ^7^ ^]^ Because of these unique features, PTAs have been used in wearable devices^[^ ^8^ ^]^ and in vivo virus inactivation.^[^ ^9^ ^]^ For example, Cai reported a strategy involving binding neutralizing antibodies onto the surface of a photothermal NP to actively capture SARS‐CoV‐2 and kill the virus upon red‐light irradiation.^[^ ^9b^ ^]^ Recently, the PIT of RABV based on PTT has achieved breakthrough results by Zhao et al., using aptamer‐conjugated RVG‐Apt‐PEG‐silica gold nanorods to target RABV in the brains of mice to treat infected mice, which played a guiding role in the treatment of neurotropic viruses.^[^ ^6e^ ^]^ However, the use of multiple injections and irradiation times increases the complexity of PEP treatment procedures. Simplifying the treatment procedure and improving the therapeutic effect are major challenges for rabies PEP and PIT.

It has been reported that photothermal effects can induce the release of inflammatory cytokines and immunogens at the irradiation site, contributing to immune stimulation.^[^ ^10^ ^]^ Inflammatory factors such as tumor necrosis factor alpha (TNF‐*α*), interferon‐*γ* (IFN‐*γ*), and interleukins (ILs) can affect antigen presentation and play an important role in natural immunity.^[^ ^11^ ^]^ Studies have shown that RABV strains can prevent the entry of immune factors by regulating the permeability of the BBB, while mild inflammatory reactions can effectively eliminate a portion of RABV in the CNS.^[^ ^12^ ^]^ Overexpression of TNF‐*α* in recombinant RABV can cause T‐cell infiltration and microglial activation through brain inflammation improving the survival rate of mice.^[^ ^13^ ^]^ Therefore, the use of a photothermal‐induced immune response to achieve synergistic antiviral effects, which can result in a simplified treatment process and reliable treatment outcomes, has attracted our interest.

**Figure 1:**
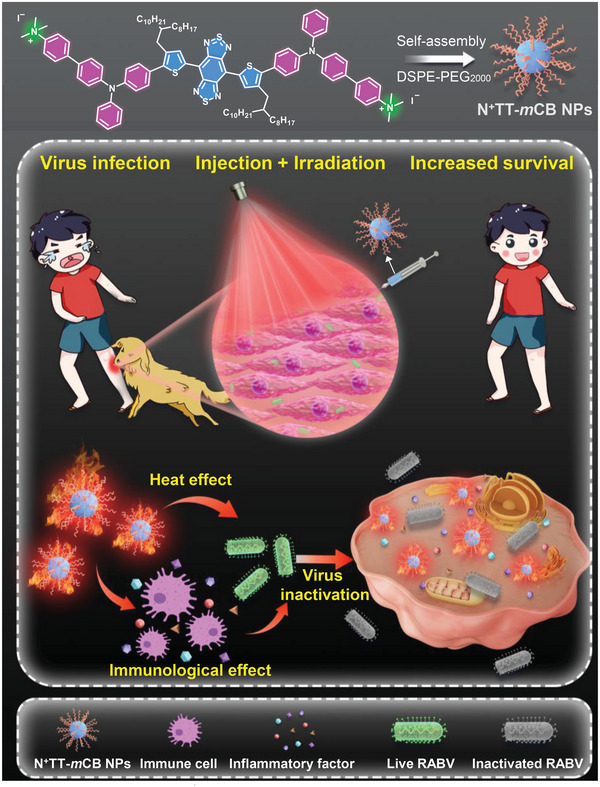
Chemical structure of N^+^TT‐*m*CB and a schematic diagram of the antiviral effects of N^+^TT‐*m*CB.

Herein, we proposed a facile strategy to realize effective PEP and PIT simultaneously by PTT and photothermally triggered immunological effects. The designed photothermal agent (N*^+^* TT‐*m*CB nanoparticles) featured positively charged functional groups and high photo‐to‐heat efficiency, which are favorable for RABV targeting and inactivation. The survival ratio of RABV‐infected mice increased after treatment with orthotopic PTT through the inhibition of local viruses via thermal effects and inflammatory factors, including TNF‐*α*, IFN‐*β* and ILs (**Figure** **1**). Interestingly, when the virus was transported to the brain, the mice were also protected by intracranial (i.c.) injection of N^+^TT‐*m*CB and irradiation. Overall, the results suggested that N^+^TT‐*m*CB with irradiation can serve as both an effective PEP and PIT, opening a new avenue for future antiviral studies.

## 2 Results and Discussions

### 2.1 Molecular Design and Synthesis

To improve the therapeutic effect, it is essential to develop high‐performance PTAs, which are photothermal therapy's cornerstone and critical components. Inorganic nanomaterials such as carbon nanomaterials and metal nanomaterials, have been tremendously utilized as PTAs.^[^ ^10^, ^14^ ^]^ In contrast, organic materials have recently gained wide attention owing to the merits of biocompatibility and biodegradability.^[^ ^15^ ^]^ The conventional strategies for designing near‐infrared (NIR)‐absorbing organic PTAs relied on the construction of strong donor (D) and acceptor (A) units into coplanar structures to enhance the non‐radiative decay in the aggregate state.^[^ ^15^, ^16^ ^]^ To further elevate the photothermal conversion efficiency (PCE), molecular rotors were grafted into NIR‐based PTA owing to the boosted nonradiative decay by activated intramolecular motions.^[^ ^17^ ^]^ These strategies have yielded marked success; however, there is still an urgent need to develop a more efficient PTA for more efficient treatment.

Herein, we proposed a highly efficient PTA designing strategy: introducing ionic groups into strong D–A systems with molecular rotors. As shown in Figure 1, the as‐prepared organic PTA (N^+^TT‐*m*CB) comprises of three key elements. First, to drive the absorption to the near infrared region, a strong electronic acceptor benzobisthiadiazole (BBTD) with a large planar *π*‐conjugated structure and electronic donor triphenylamine (TPA) were selected as the molecular skeleton. The planar thiophene (TP) unit was utilized as a *π*‐bridge to facilitate the intramolecular charge transfer from TPA to BBTD. Second, the long‐branched alkyl chains at the meta position of thiophene provided some necessary room to promote free intramolecular motion for enhanced nonradiative decay. Third, a cationic trimethylammonium unit was introduced to boost D–A interactions for further enhanced nonradiative decay (heat generation). Meanwhile, the cationic units are beneficial for improving the interaction with negatively charged enveloped viruses through charge interactions. The synthetic routes to N^+^TT‐*m*CB and other involved molecules are presented in Figure S1, Supporting Information. These molecules were fully characterized by NMR spectroscopy and high‐resolution mass spectrometry (Figures S2–S10, Supporting Information).

**Figure 2:**
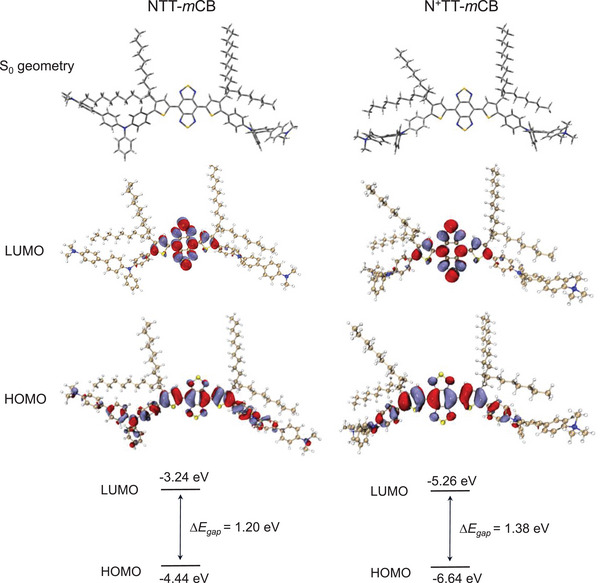
Theoretical calculation analysis. The optimized S0 geometry, HOMO, LUMO, and energy gap of NTT‐*m*CB and N^+^TT‐*m*CB.

To better embody the photophysical properties of N^+^TT‐*m*CB, NTT‐*m*CB without a positive charge was used as a control. The electronic structures in the ground state (S0) determined at the B3LYP/6G(d) level of the theory suggest that both NTT‐*m*CB and N^+^TT‐*m*CB exhibit a planar structure with a dihedral angle between BBTD and TPA of ≈1° (**Figure** **2**). Their highest occupied molecular orbitals (HOMO) were delocalized by the conjugated backbone, whereas the lowest unoccupied molecular orbitals (LUMO) were restricted to the TP‐BBTD‐TP core (Figure 2). The narrow energy gap (Δ*E*_gap_) between highest occupied molecular orbitals (HOMO) and lowest unoccupied molecular orbitals (LUMO) for NTT‐*m*CB and N^+^TT‐*m*CB (1.20 and 1.38 eV, respectively) was beneficial for strong absorptivity in the NIR biological window. The slightly larger Δ*E*_gap_ of N^+^TT‐*m*CB was owing to the weakening of the electron‐donating ability of TPA by the electron‐deficient trimethylammonium unit.

### 2.2 Photophysical Properties

**Figure 3:**
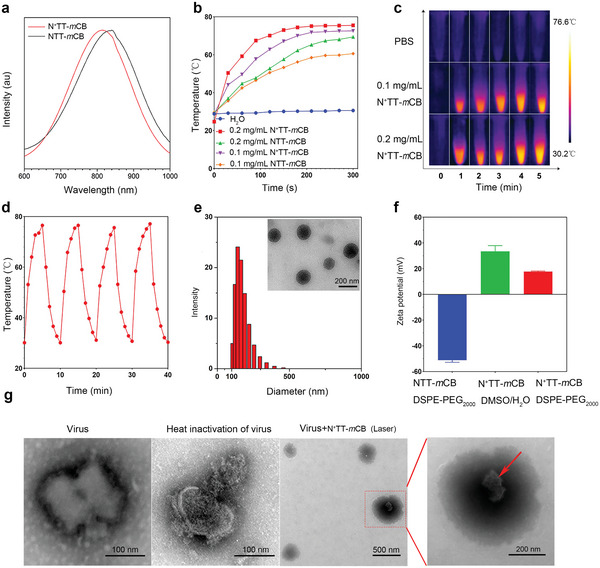
Characterization of N^+^TT‐*m*CB. a) Normalized absorption spectrum of N^+^TT‐*m*CB and NTT‐*m*CB (wavelength, 600–1000 nm). b) Temperature rise curves of N^+^TT‐*m*CB and NTT‐*m*CB NPs with varying concentrations (0.1 mg mL^−1^, 0.2 mg mL^−1^) under 808 nm, 0.45 W cm^−2^ irradiation. c) Thermographic images of N^+^TT‐*m*CB NPs at various times (1–5 min) and concentrations (0.1 mg mL^−1^, 0.2 mg mL^−1^) under 808 nm, 0.45 W cm^−2^ NIR irradiation. d) Cyclic photothermal heating and cooling of 0.2 mg mL^−1^ N^+^TT‐*m*CB under 0.45 W cm^−2^ NIR irradiation for 5 min (repeated four times). e) DLS data and TEM image of N^+^TT‐*m*CB NPs. f) Zeta potentials of NTT‐*m*CB‐DSPE‐PEG_2000_, N^+^TT‐*m*CB‐DMSO/H_2_O, and N^+^TT‐*m*CB‐DSPE‐PEG_2000_. g) TEM images of live viruses, heat‐inactivated viruses (65 °C, 10 min), and viruses commixed with N^+^TT‐*m*CB under 808 nm, 0.45 W cm^−2^ irradiation for 5 min. The red arrow indicates the structure of a virus that was destroyed after PTT.

The absorption properties of N^+^TT‐*m*CB and NTT‐*m*CB were characterized by UV–vis–NIR spectroscopy. As shown in **Figure** **3**a, N^+^TT‐*m*CB and NTT‐*m*CB displayed strong NIR absorption in the spectral region of 600–1000 nm with peaks located at 810 and 838 nm, respectively, which demonstrated a huge potential for NIR light‐triggered photothermal theranostic. The blue‐shifted maximum absorption of N^+^TT‐*m*CB compared with NTT‐*m*CB is mainly owing to the introduction of an electron‐deficient trimethylammonium unit that weakens the electron‐donating ability of TPA, giving decreased D–A effects. Moreover, the larger Δ*E*_gap_ of N^+^TT‐*m*CB also explained its blueshifted wavelength. To analyze these materials in the aggregate state, the NPs were prepared by nanoprecipitation using 1,2‐distearoyl‐sn‐glycero‐3‐phosphoethanolamine‐*N*‐[methoxy‐(polyethylene glycol)‐2000] (DSPE‐PEG_2000_) as the encapsulation matrix. Similarly, the absorption peaks of N^+^TT‐*m*CB NPs (≈810 nm) were at shorter wavelengths than those of NTT‐*m*CB (Figure S11, Supporting Information). The molar absorption of N^+^TT‐*m*CB NPs was calculated to be 2.05 × 10^4^ L mol^−1^cm^−1^ at 808 nm. Such strong and long‐wavelength absorption indicated that N^+^TT‐*m*CB is especially suitable for application as a photothermal therapeutic agent.

Motivated by the strong NIR absorption, we sought to investigate the photothermal conversion ability of these PTAs. The temperature change of N^+^TT‐*m*CB and NTT‐*m*CB NPs was recorded under laser irradiation with an NIR laser (808 nm, 0.45 W cm^−2^) for 5 min. As shown in Figure 3b,c, the temperature of the N^+^TT‐*m*CB NPs gradually increased in a concentration‐dependent and time‐dependent manner. The maximum temperatures of N^+^TT‐*m*CB after 5 min increased to 75.5 (0.2 mg mL^−1^) and 72.6 °C (0.1 mg mL^−1^), better than that of NTT‐*m*CB (69.4 and 60.6 °C for 0.2 and 0.1 mg mL^−1^, respectively). The increased photothermal conversion ability of N^+^TT‐*m*CB NPs was attributed to the enhanced nonradiative decay for heat generation owing to the introduction of cationic trimethylammonium unit. The PCE was calculated to ≈78%, which is comparable to other reported organic PTAs (Figure S12, Supporting Information).^[^ ^18^ ^]^ Moreover, even after four cycles of heating‐to‐cooling, the N^+^TT‐*m*CB NPs can still recover to its maximum temperature suggesting the high photostability (Figure 3d).

The N^+^TT‐*m*CB NPs are spherical, have good water dispersibility and have a diameter of ≈144 nm, as determined by transmission electron microscopy (TEM) and dynamic light scattering (DLS) (Figure 3e). Furthermore, we observed no obvious precipitation during storage at room temperature for at least 144 h in PBS and Dulbecco's modified Eagle's medium (DMEM, Figure S13, Supporting Information), indicating that N^+^TT‐*m*CB NPs possessed excellent colloidal stability.

### 2.3 Antiviral Effect of N^+^TT‐*m*CB In Vitro

The phospholipid bilayer on the surface of most enveloped viruses is negatively charged, so positively charged molecules are more likely to interact with the virus and further improve the therapeutic effect. Although the N^+^TT‐*m*CB NPs were encapsulated by DSPE‐PEG_2000_, the zeta potential of N^+^TT‐*m*CB NPs remained at +17.67 mV (Figure 3f), which is beneficial for virus binding. To confirm the binding efficiency and virus inactivation ability using PTT, transmission electron microscopy (TEM) was conducted to investigate the morphology of the virus. Normal virus particles are bullet‐shaped with a visible vesicle and tightly packed internal proteins, whereas heat‐inactivated virus particles are sparsely packed with proteins.^[^ ^19^ ^]^ Moreover, it was found that virus particles could be detected on the surface of N^+^TT‐*m*CB, the vesicle structure of the virus particles was disrupted, and the internal protein structure was sparse (Figure 3g). This is similar to the Das research, inactivation of the capsid virus results in disintegration of the capsid membrane and release of the contents. All these results suggested that N^+^TT‐*m*CB NPs can interact with viruses and kill them by photothermal effects due to their hydrophilic positive charges and high PCE. Moreover, the viability of BSR cells treated with N^+^TT‐*m*CB NPs indicated the negligible cytotoxicity of these NPs (Figure S14, Supporting Information), demonstrating their good biocompatibility.

**Figure 4:**
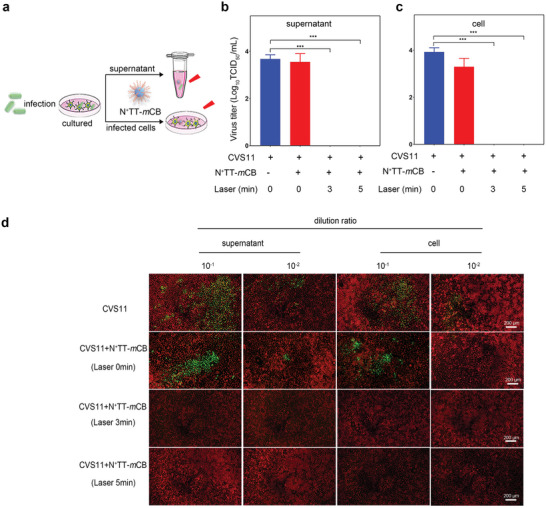
Antiviral effect of N^+^TT‐*m*CB in vitro. a) Flow chart of the N^+^TT‐*m*CB antiviral effect in vitro. b,c) RABV titer in the supernatant and cells with different treatments. d) Representative fluorescence images of RABV in the supernatant and cells at different dilution ratios. Green represents virus‐infected cells and red represents cells without virus infection. The cells were strained with FITC anti‐rabies monoclonal globulin (1:200) and Evans blue (1:500). Scale bar (200 µm). Bar graph represents means ± SD, *n* = 3. Statistical analysis of grouped comparisons was carried out by means of Student's *t* test (***p < 0.001).

Considering the high photothermal performance and good biocompatibility of N^+^TT‐*m*CB, its antiviral ability on cells was evaluated. Cell supernatant containing RABV CVS11 and cells infected with RABV were treated with N^+^TT‐*m*CB and then irradiated with an NIR laser (808 nm, 0.45 W cm^−2^) for 0, 3, or 5 min. The experimental process is shown in **Figure** **4**a. Surprisingly, we found that the virus titer in the supernatant and cells decreased significantly after 3 min of irradiation, and even the green fluorescent spots of the virus could not be detected (Figure 4b–d). Next, we used the RABV vaccine strain SRV9 to verify that N^+^TT‐*m*CB was equally effective in killing the virus against a different strain. Similarly, the virus titer in the cell culture supernatant decreased significantly after 3 min of irradiation, and we could not detect the virus inside the RABV‐infected cells after 3 min of irradiation (Figure S15, Supporting Information). These results indicated that the PTT effect of N^+^TT‐*m*CB upon laser irradiation exhibited effective antiviral ability in vitro.

### 2.4 Antiviral Effect of N^+^TT‐*m*CB In Vivo

**Figure 5:**
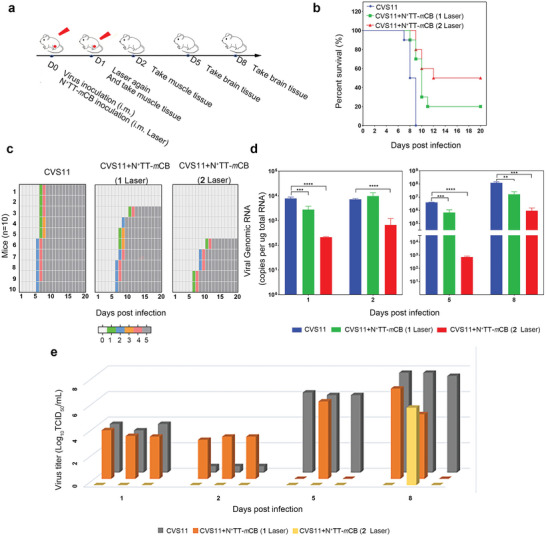
Therapeutic effect of N^+^TT‐*m*CB‐based PTT in vivo. a) Experimental flow chart of the therapeutic effect of N^+^TT‐*m*CB‐based PTT in mice. b) Mortality rates of mice (control, N^+^TT‐*m*CB NPs +1 laser, and N^+^TT‐*m*CB NPs +2 laser, *n* = 10) were recorded daily for 20 days. c) Clinical score of the three groups of mice mentioned above (*n* = 10). d) Copies of the RABV N gene of mice (*n* = 3) were determined by qPCR. The muscles were tested at 1 dpi and 2 dpi, while the brains were tested at 5 dpi and 8 dpi. e) The virus titers of the mice mentioned above were determined by TCID_50_ (*n* = 3). The muscles were tested at 1 dpi and 2 dpi, while the brains were tested at 5 dpi and 8 dpi. Bar graph represents means ± SD. Statistical analysis of grouped comparisons was carried out by Student's *t* test (**p < 0.01, ***p < 0.001, ****p < 0.0001).

To assess the antiviral effect of N^+^TT‐*m*CB‐based PTT in vivo, the experimental procedure is shown in **Figure** **5**a. Six‐week‐old ICR mice were inoculated with RABV‐CVS11 at 5 × 10^5^ FFU by i.m. injection and then randomly divided into 3 groups (control, N^+^TT‐*m*CB NPs+1 laser, and N^+^TT‐*m*CB NPs+2 laser, *n* = 22). The N^+^TT‐*m*CB NPs treatment groups were immediately injected with 0.2 mg mL^−1^ N^+^TT‐*m*CB NPs and irradiated with NIR laser (808 nm, 0.45 W cm^−2^) for 5 min. The twice‐irradiated group was irradiated for 5 min again 24 h later without NPs injection. The survival rate of the mice was increased in the treatment group and reached 50% after two rounds of irradiation, while all mice in the control group died (Figure 5b). Moreover, the clinical score results showed that the mice in the RABV group began to show clinical symptoms on day 6, while mice in the N^+^TT‐*m*CB NPs+1 laser and N^+^TT‐*m*CB NPs+2 laser groups had fewer symptoms than those in the control group (Figure 5c).

Then, to clarify the reasons for the increased mortality, we first quantitatively analyzed the RABV load in different tissues. Hindlimb muscle tissue and brain tissue were collected on different days and subjected to qPCR and virus titer measurements. The qPCR results showed that the viral load at the site of infection was significantly reduced after N^+^TT‐*m*CB NP + laser irradiation. In particular, the virus titer could not be detected after 2 rounds of laser irradiation. These results suggest that the N^+^TT‐*m*CB NP+2 laser treatment can adequately inactivate the virus and play a protective role in mice (Figure 5d,e). Importantly, the virus copies in the brains of the mice in the treatment groups (N^+^TT‐*m*CB NPs+1 laser and N^+^TT‐*m*CB NPs+2 laser) were significantly lower than those in the control group. Notably, the virus was detected in only one mouse on the 8th day. Overall, these results show that the virus can be actively killed at the infection site and that the virus transfer to the brain was reduced through one N^+^TT‐*m*CB NP injection and two laser irradiation treatments. The simple operation and effective prevention provide new ideas for the PEP of RABV.

**Figure 6:**
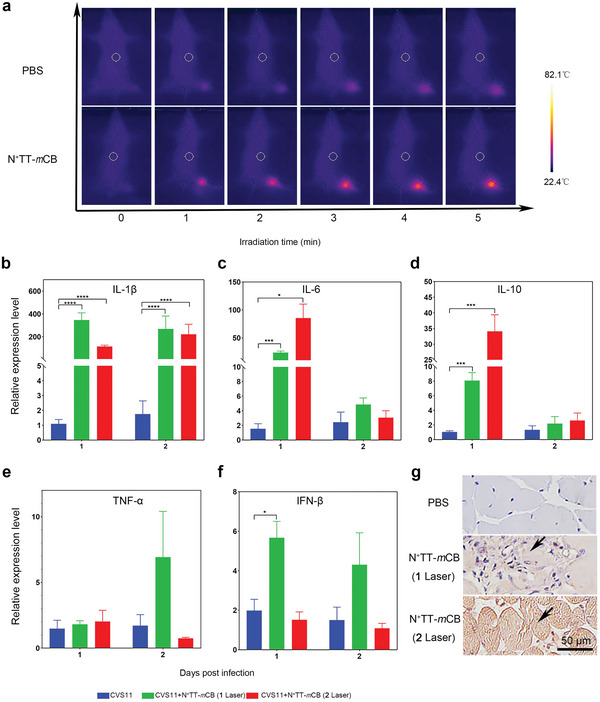
Changes in temperature and cytokines after i.m. injection of N^+^TT‐*m*CB with laser irradiation (808 nm, 0.45 W cm^−2^) for 5 min in mice. Six‐week‐old ICR mice were infected with RABV at 5 × 10^5^ FFU by i.m. injection at 0 dpi, and the N^+^TT‐*m*CB NP treatment groups were immediately injected with 0.2 mg mL^−1^ N^+^TT‐*m*CB NPs and irradiated with NIR (808 nm, 0.45 W cm^−2^) for 5 min. The twice‐irradiated group was irradiated for 5 min again 24 h later without NP injection. a) The temperature changes of mice after i.m. injection of PBS and N^+^TT‐*m*CB (0.2 mg mL^−1^), followed by laser irradiation (808 nm, 0.45 W cm^−2^) for 5 min. b–f) The cytokine changes in muscle tissue from mice subjected to different treatments (*n* = 3). g) Inflammation (IL‐6) in muscle was determined by IHC. The muscle sections were visualized upon incubation with mouse anti‐IL‐6 antibody (1:400) and HRP‐conjugated goat anti‐mouse antibody (1:5000). The black arrows represent the produce of IL‐6 after irradiation. Scale bar (50 µm). Bar graph represents means ± SD. Statistical analysis of grouped comparisons was carried out by Student's *t* test (*p < 0.05, ***p < 0.001, ****p < 0.0001).

To elucidate the mechanism of virus reduction, we monitored the temperature change and inflammatory cytokines of mice at the irradiation site. The temperature reached as high as 60–70 °C after intramuscular injection of N^+^TT‐*m*CB and laser irradiation, which is favorable for virus inactivation (**Figure** **6**a). Meanwhile, N^+^TT‐*m*CB cannot produce •OH and 1O_2_, which means the photothermal rather than photodynamic effect dominate the virus inactivation ability (Figure S16, Supporting Information). Moreover, it has been reported that PTT often damages the integrity of cell membranes and causes the release of intracellular components, leading to proinflammatory reactions.^[^ ^20^ ^]^ Studies have found that release cytokines such as TNF‐*α*, IFN‐*γ*, and ILs contribute to the control or elimination of viral infections by activating IFN‐stimulated genes to exert direct antiviral effects and recruit antiviral immune effector cells to clear the virus.^[^ ^21^ ^]^ Inflammatory factors play an important role in the process of RABV infection.^[^ ^22^ ^]^ Thus, we speculated that in addition to heat, inflammatory cytokines induced by heat may also play a role in the antiviral process. The integrity of cells was damaged with propidium iodide entering cells and exhibiting red fluorescence in Figure S17, Supporting Information, which is necessary for the proinflammatory reaction. Then, we compared the inflammatory cytokines in the hindlimb muscle tissues of mice subjected to different treatments. The results showed that a range of inflammatory factors, including IL6, IL‐10 and IL‐1*β*, were produced in mice after PTT, even after only 1 round of laser irradiation (Figure 6b–f). Excessive secretion of IL‐6 was detected in the N^+^TT‐*m*CB + laser irradiation site, while no inflammatory factors were produced in the nonirradiated sites, as determined by immunohistochemistry (IHC) analysis (Figure 6g). In particular, TNF‐*α*, IL‐1, and IL‐6 participated in macrophage activation and induced an early innate immune response. Therefore, not only the photothermal effect of N^+^TT‐*m*CB but also the expression of a large number of inflammatory factors under heat stimulation together led to the reduction of virus in the infected site, which was also an important reason for the survival of mice.

**Figure 7:**
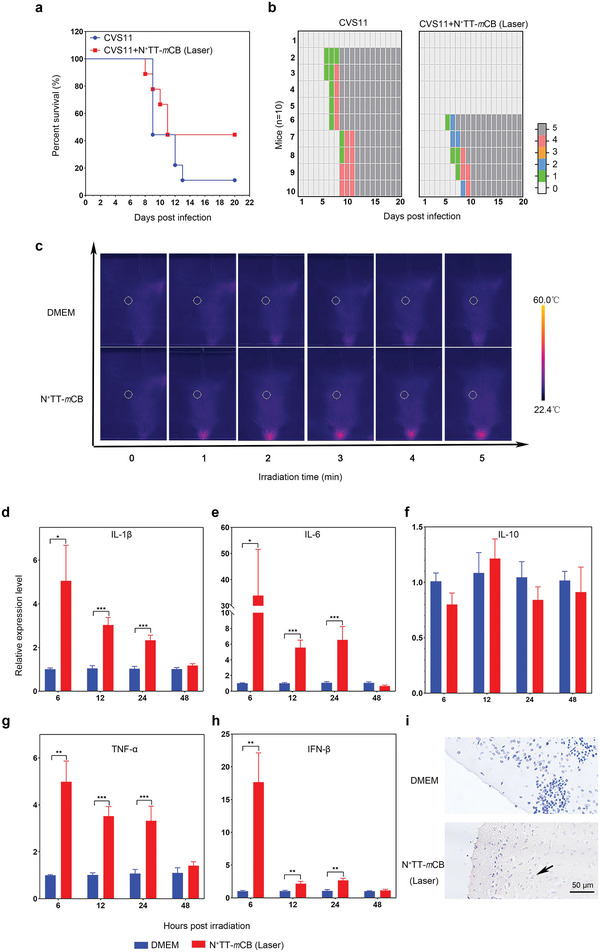
Therapeutic effect of N^+^TT‐*m*CB‐based PTT in vivo. a) Mortality rates of mice (RABV without N^+^TT‐*m*CB treatment, RABV with N ^+^ TT‐*m*CB laser, *n* = 10) were recorded daily for 20 days. b) Clinical score of the mice mentioned above (*n* = 10). c) The temperature changes of mice after i.c. injection of PBS and N^+^TT‐*m*CB (0.2 mg mL^−1^) and irradiated with NIR (808 nm, 0.45 W cm^−2^) for 5 min. d–h) The cytokine changes in mice after i.c. injection of PBS and N^+^TT‐*m*CB (0.2 mg mL^−1^) and irradiation with NIR (808 nm, 0.45 W cm^−2^) for 5 min (*n* = 3). Bar graph represents means ± SD. Statistical analysis of grouped comparisons was carried out by Student's *t* test (*p < 0.05, **p < 0.01, ***p < 0.001). i) Brain inflammation (IL‐6) was determined by IHC. The brain sections were visualized by incubation with mouse anti‐IL‐6 antibody (1:400) and HRP‐conjugated goat anti‐mouse antibody (1:5000). The black arrow represents the preduce of IL‐6 after irradiation. Scale bar (50 µm).

Since strong inflammatory factors were secreted upon one instance of heat stimulation, we wondered if PTT of the brain would have a therapeutic effect on infected mice. It has been reported that inflammatory cytokines (IL‐6 and IL‐1*β*) can activate astrocytes, which results in the release of angiogenic factors, thus increasing BBB permeability, affecting the passage of cytokines and chemokines and regulating immune responses. Therefore, we conducted a therapy experiment with mice inoculated with 3 × 10^5^ FFU RABV‐CVS11 virus in the hind limb muscles. N^+^TT‐*m*CB was injected intracranially after 3 days of infection, and the mice were subjected to 5 min of laser irradiation (808 nm, 0.45 W cm^−2^). Surprisingly, the survival rate was increased by 40% in the N^+^TT‐*m*CB + laser group compared with the control group. Clinical symptoms of the mice were observed daily, and the results showed that the clinical score of the mice in the treated group was lower than that of the mice in the control group (**Figure** **7**a,b). These results suggest that PTT can kill viruses and achieve therapeutic efficacy in mice after 3 days of infection. To decipher the therapeutic mechanism, the temperature of the irradiated site was recorded after intracranial injection of N^+^TT‐*m*CB NPs. As shown in Figure 7c, the temperature reached ≈55–60 °C after 5 min of irradiation. At the same time, inflammatory factors were detected in the brain tissues of mice at 6, 12, 24, and 48 h after irradiation. The results showed that IL‐1*β*, TNF‐*α*, IL‐6, and IFN‐*β* were highly expressed within 24 h after irradiation (Figure 7d–h). The IHC results showed that the irradiated brain tissue was brown, which indicated the production of inflammatory factors at the irradiation site, suggesting that the survival of mice might be related to PTT and its induced inflammatory factors (Figure 7i). In conclusion, we realized PIT after viral infection through the heat and immunological effects induced by photothermal reagents.

### 2.5 Safety Assessment of N^+^TT‐*m*CB In Vivo

**Figure 8:**
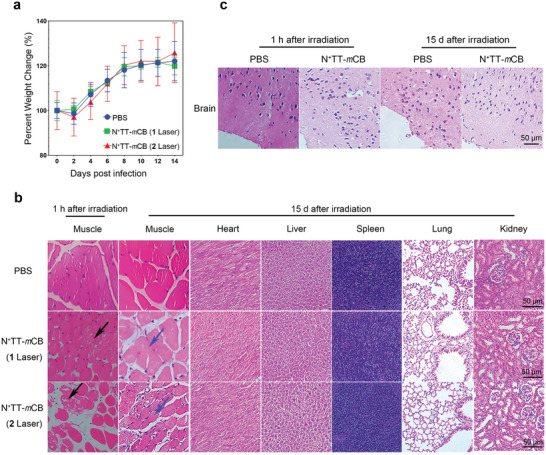
Safety assessment of N^+^TT‐*m*CB in mice. a) The body weight changes of the mice with different treatments (PBS, N^+^TT‐*m*CB irradiated once, N^+^TT‐*m*CB irradiated twice) recorded for 14 days (*n* = 10). Bar graph represents means ± SD. Statistical analysis of grouped comparisons was carried out by Student's *t* test. b,c) H&E‐stained images of tissue slices of major organs (muscle, heart, liver, spleen, lung, kidney, and brain) of mice in different groups. The black arrows represent the presence of muscle damage after irradiation and the blue arrows represent muscle recovery at 15 d after irradiation. Scale bar = 50 µm.

Finally, the long‐term toxicity of N^+^TT‐*m*CB NPs was investigated. Six‐week‐old ICR mice were i.m. injected with PBS or 0.2 mg mL^−1^ N^+^TT‐*m*CB and irradiated one or twice with NIR light (808 nm, 0.45 W cm^−2^) for 5 min. We assessed the safety by monitoring body weight changes and performing routine blood analysis, measuring biochemical indexes and analyzing tissue sections. The results showed that the body weight (**Figure** **8**a), routine blood analysis and biochemical indexes were all within the normal ranges, regardless of whether the mice were irradiated once or twice by NIR (Table S1, Supporting Information). Their organs and tissues (heart, liver, spleen, lung, and kidney) showed no obvious cellular damage. Next, we examined the pathological changes at the irradiated site by H&E staining, and the results showed that the internal structure of the irradiated muscle fibers was disorganized and that the myocytes were scattered in the interstitium in a pathological state, Fortunately, H&E staining showed some recovery of the irradiated muscle state relative to the irradiated muscle after 15 days (Figure 8b). The H&E staining results of a brain with PTT showed that the nuclei of the irradiated nerve cells were deeply stained and that the nuclei were crinkled, while there was some restoration of nerve cells at the irradiated site after 15 d (Figure 8c). Thus, although the laser irradiation would induce local small‐scale damage, it is fortunate that it will be repaired locally, which demonstrated that N^+^TT‐*m*CB can be used as a safe biological material for RABV PEP and PIT.

## 3 Conclusion

In summary, we designed a novel PTA (N^+^TT‐*m*CB) with strong NIR absorption, high PCE and effective antivirus ability by introducing strong *π*‐conjugation, molecular rotors and hydrophilic positive charges. By applying the principle of mutual attraction of positive and negative charges, the NPs capture the virus and further kill the virus via PTT. Because of the high antiviral ability, we proposed a simple and effective PEP strategy involving immediate injection of N^+^TT‐*m*CB NPs at the site of infection followed by irradiation. The survival rate of RABV‐infected mice increased by inhibiting local viruses via thermal effects and photothermal triggered inflammatory reactions, including TNF‐*α*, IFN‐*β* and ILs. Interestingly, when the virus was transported to the brain, the mice were protected by intracranial (i.c.) injection of N^+^TT‐*m*CB and irradiation. Overall, the results suggested that N^+^TT‐*m*CB + irradiation can serve as not only an effective PEP strategy but also a postinfection treatment method. The photothermal therapy proposed in this study provides a potential method for future antiviral research.

## 4 Experimental Section

### Viruses, Cells, Antibodies, and Animals

Viruses (CVS11 strain, SRV9‐eGFP strain) were preserved in the authors’ lab. The virus was propagated, titered by N2a cells and stored in a −80 °C refrigerator. N2a (mouse neuroblastoma) and BSR (cloned from BHK‐21) cells were cultured in DMEM (GIBCO, USA) supplemented with 10% fetal bovine serum (GIBCO, USA), 100 units/mL penicillin and 100 µg mL^−1^ streptomycin sulfate. FITC anti‐rabies monoclonal globulin (Fujirebio, USA). IL‐6 monoclonal antibody (Proteintech, USA). All animals were used according to the protocol of the Scientific Ethics Committee of Chinese laboratory animals. The agreement was approved by the Animal Welfare and Ethics Committee of the Institute of Veterinary Medicine of the Changchun Veterinary Research Institute (Laboratory Animal Care and Use Committee Authorization, permit number IACUC of AMMS‐11‐2022‐041).

### Virus Titration

Virus titration assays were performed using N2a cells in a direct fluorescent antibody (DFA) assay. In brief, N2a cells were incubated with tenfold serial dilutions of the virus supernatant at 37 °C for 48 h in a 96‐well cell culture plate in quadruplicate. The cells were fixed with 80% cold acetone for 30 min at room temperature and then washed three times with phosphate‐buffered saline (PBST). Then, the cells were stained with FITC anti‐rabies monoclonal globulin (1:200) and Evans blue (1:500) for 1 h at 37 °C and washed three times with PBST. The positive spots were observed by fluorescence microscopy and the Karber method was used to calculate virus titers.

### Antiviral Effect of N^+^TT‐*m*CB In Vitro

BSR cells were cultured in 96‐well plates and infected with CVS11 and SRV9‐eGFP at MOI = 0.1 for 1 h at 37 °C. Then, the liquid was discarded and fresh medium was added to continue the culture for 24 h at 37 °C. After 24 h of cultivation, the cell supernatant was added to the same volume of 0.2 mg mL^−1^ N^+^TT‐*m*CB NPs and the infected cells were incubated with 150 µL of DMEM containing N^+^TT‐*m*CB NPs (the final concentration was 0.1 mg mL^−1^). They were irradiated with NIR light (808 nm, 0.45 W cm^−2^) for 0, 3, or 5 min. Subsequently, the irradiated supernatants and the infected cells were separately collected for the detection of virus titers by TCID_50_ assay. Fluorescence was observed under a fluorescence microscope (Olympus, Japan).

### Safety Assessment of N^+^TT‐*m*CB NPs in Mice

Six‐week‐old female ICR mice (*n* = 10) were randomly divided into 3 groups (PBS, 0.2 mg mL^−1^ N^+^TT‐*m*CB irradiated once and 0.2 mg mL^−1^ N^+^TT‐*m*CB NPs irradiated twice) and inoculated with PBS and N^+^TT‐*m*CB by intramuscular (i.m.) injection, respectively. The irradiation groups were irradiated with NIR (808 nm, 0.45 W cm^−2^) for 5 min at the injection site. The twice‐irradiated group received the same treatment 24 h later. After irradiation, the muscle tissues were removed for H&E staining and IHC. Mouse body weights were recorded every other day for a total of 15 days. The blood and organs of the mice were collected 15 days after irradiation. The blood was used for the routine blood analysis and measurement of biochemical indexes. Organs (heart, liver, spleen, lung, kidney, and muscle) were fixed for H&E staining and IHC. Moreover, six‐week‐old female ICR mice were inoculated with PBS and N^+^TT‐*m*CB NPs by intracranial (i.c.) injection and irradiated with NIR (808 nm, 0.45 W cm^−2^) for 5 min. The brain tissues were removed for H&E staining or IHC immediately after irradiation and 15 days after irradiation, respectively.

### Antiviral Effect of N^+^TT‐*m*CB In Vivo

Six‐week‐old female ICR mice (*n* = 22) were randomly divided into 3 groups (control, N^+^TT‐*m*CB NPs+1 laser and N^+^TT‐*m*CB NPs+2 laser) and were i.m. injected with 5 × 10^5^ FFU RABV‐CVS11. The N^+^TT‐*m*CB NP treatment groups were immediately injected with 0.2 mg mL^−1^ N^+^TT‐*m*CB and irradiated with NIR (808 nm, 0.45 W cm^−2^) for 5 min. The twice‐irradiated group received the same irradiation 24 h later without NP injection. The clinical symptoms and mortality were observed once a day for 20 days. The evaluation of clinical symptoms adopts the scoring system, that is, 0 indicates no symptoms; 1 point indicates uncoordinated movement; 2 points indicates rough coat and arched back; 3 points represent tremor and mania; 4 points represent ataxia and paralysis; and a score of 5 indicates death. Mouse brain and muscle tissues were separately collected at 1, 2, 5, and 8 days after irradiation (*n* = 3) and were used to detect the viral load as well as the expression of inflammatory factors.

### Postinfection Treatment

Six‐week‐old ICR mice (*n* = 10) in 2 groups (control, N^+^TT‐*m*CB NPs+1 laser) were inoculated with RABV‐CVS11 at 3 × 10^5^ FFU by i.m. injection. At 3 days postinfection, the N^+^TT‐*m*CB group was inoculated with 0.2 mg mL^−1^ N^+^TT‐*m*CB by i.c. injection and irradiated with NIR (808 nm, 0.45 W cm^−2^) for 5 min. The clinical symptoms and mortality were observed once a day for 20 days.

### Statistical Analysis

All the quantitative data in each experiment are presented as the mean values ± SD. The sample size is provided separately in the figure caption. The statistical significance was determined using Student's *t* test. Values of *p < 0.05, **p < 0.01, ***p < 0.001, and **** *p* < 0.0001 indicated statistically significant differences. All data were analyzed using GraphPad Prism 8.

## Conflict of Interest

The authors declare no conflict of interest.

## Supporting information

Supporting Information

## Data Availability

The data that support the findings of this study are available from the corresponding author upon reasonable request.
