# Supplementary material for: Treat the “Untreatable” by a Photothermal Agent: Triggering Heat and Immunological Responses for Rabies Virus Inactivation
Source: Adv Sci (Weinh). 2022 Nov 17;10(2):2205461. doi: 10.1002/advs.202205461 (PMC9839883; doi:10.1002/advs.202205461)
Supplement: Supplementary file 1 — Supporting Information [file ADVS-10-2205461-s001.pdf]

ADVANCED SCIENCE
Open Access

Supporting Information

for *Adv. Sci.*, DOI 10.1002/advs.202205461

Treat the "Untreatable" by a Photothermal Agent: Triggering Heat and Immunological Responses for Rabies Virus Inactivation

Yujie Bai, Pei Huang, Na Feng, Yuanyuan Li, Jingbo Huang, Hongli Jin, Mengyao Zhang, Jingxuan Sun, Nan Li, Haili Zhang, Xianzhu Xia, Ben Zhong Tang* and Hualei Wang**

Supporting Information

Treat the "Untreatable" by a Photothermal Agent: Triggering Heat and Immunological Responses for Rabies Virus Inactivation

Yujie Bai#, Pei Huang#, Na Feng#, Yuanyuan Li*, Jingbo Huang, Hongli Jin, Mengyao Zhang, Jingxuan Sun, Nan Li, Haili Zhang, Xianzhu Xia, Ben Zhong Tang*, Hualei Wang*

Y. Bai, Dr. P. Huang, Prof. Y. Li, J. Huang, Dr. H. Jin, M. Zhang, J. Sun, Dr. H. Zhang, Prof. H. Wang
Key Laboratory of Zoonosis Research, Ministry of Education, College of Veterinary Medicine, Jilin University, Changchun130062, China
E-mail: li_yuanyuan@jlu.edu.cn; wanghualei@jlu.edu.cn

Dr. N. Feng, N. Li, Prof. X. Xia
Changchun Veterinary Research Institute, Chinses Academy of Agricultural Sciences, Changchun130122, China

Prof. B. Z. Tang
School of Science and Engineering, Shenzhen Institute of Aggregate Science and Technology, The Chinese University of Hong Kong, Shenzhen, Guangdong 518172, China
E-mail: tangbenz@cuhk.edu.cn

#These authors contributed equally to this work

Supplementary Methods

Materials

All the chemicals and reagents were purchased from chemical sources, and the solvents for chemical reactions were distilled before use. Benzo[1,2-c:4,5-c′] bis ([1,2,5] thiadiazole) was purchased from Derthon Optoelectronic Materials Science Technology Co LTD. All air- and moisture-sensitive reactions were carried out in flame-dried glassware under a nitrogen atmosphere.

Measurements

^{1}H and ^{13}C NMR spectra were recorded at room temperature on a Unity-400 NMR spectrometer using $CDCl_3$ or DMSO as solvents and tetramethylsilane as a reference. Mass spectrometry (MS) was conducted with a GCT premier CAB048 mass spectrometer in matrix-assisted laser desorption-ionization time-of-flight (MALDI-TOF) mode. Dynamic light scattering (DLS) was measured on a 90 plus particle size analyser. Transmission electron microscopy (TEM) images were acquired on a JEM-2010F transmission electron microscope with an accelerating voltage of 200 kV. Density functional theory (DFT) calculations were carried out at the B3LYP/6G(d) level of theory by applying the Gaussian 09 package.

Preparation of N^+TT-*m*CB NPs

A mixture of N^+TT-*m*CB (1 mg), DSPE-PEG2000 (2 mg), and THF (1 mL) was sonicated (12 W output, XL2000, Misonix Incorporated, NY) to obtain a clear solution. The mixture was quickly injected into water (9 mL), which was sonicated vigorously in water for 2 min. The mixture was stirred in a fume hood under N_2 bubbling overnight to remove the THF. N^+TT-*m*CB NPs were subjected to ultrafiltration (molecular weight cut-off of 100 kDa) at 3000 × g for 30 min.

Cell viability assay

The cytotoxicity of N^+TT-*m*CB NPs in BSR cells was determined by CCK-8 assay, which was performed according to the manufacturer’s instructions (Beyotime, China). Briefly, cells were seeded at 3×10^4 per well into 96-well plates and cultured overnight. When the cells grew to 60-70%, the medium was changed to DMEM supplemented with different concentrations of N^+TT-*m*CB NPs to continue the incubation for 24 h. Then, 10 μL CCK8 was added to each well and incubated at 37 °C for 1 h. The absorbance at 450 nm was detected with a microplate reader. The cell survival rate was calculated with the absorbance value.

RNA isolation and quantitative real-time PCR (qPCR)

The mouse brains and muscles were homogenized separately and centrifuged at 4 °C, 8000 r.p.m. for 5 min. RNA was isolated from the cell pellet using TRIzol reagent (0.1 g/ml) according to the manufacturer's instructions (TIANGEN, China) and reverse transcribed into cDNA using the PrimeScript™ RT reagent Kit for qPCR (TAKARA, Japan). The standard curve was set with the positive plasmid pcDNA3.1-RABV N. The following primers were used for qPCR: RABV-N-F, GGGGCTATTTGGGAGAGGA; RABV-N-R, ATGAGTTTGGACGGGCTTG. The copy number in 0.1 g of tissue was calculated according to the generated standard curve. The expression of inflammatory factors in tissues was detected by relative quantitative real-time PCR. The primers used for qPCR are listed in Tab. S2. All qPCR experiments were performed on a StepOnePlus Real-Time PCR System (Thermo, USA).

H&E staining and IHC

Organizations (muscle, brain) were placed in 10% formaldehyde solution and fixed for at least 24 h. The tissue was dehydrated in gradient alcohol, cleared in xylene, paraffin, and embedded. Then, the slices were dewaxed with xylene, rehydrated with 100%, 90%, 80% anhydrous ethanol in turn, put into haematoxylin staining solution for 5 min, rinsed with tap water, dehydrated with 80% alcohol and stained in eosin dye solution for 3 min; the sections were put into 80%, 90%, and 100% anhydrous ethanol I and II and xylene I and II in turn for 3 min each time, dehydrated and cleared in turn. The slices were removed, and the tissue surface was sealed with drops of neutral gum resin.

The mouse muscles and brains were fixed in 10% formaldehyde solution. The fixed brains were embedded in paraffin wax and cut into 4-5 μm-thick sections. After antigen retrieval in citric acid buffer, block endogenous peroxidase in 30% hydrogen peroxide for 20 min in IHC. The mouse brain sections were incubated with mouse anti-IL-6 antibody to detect inflammation.

Supporting figures

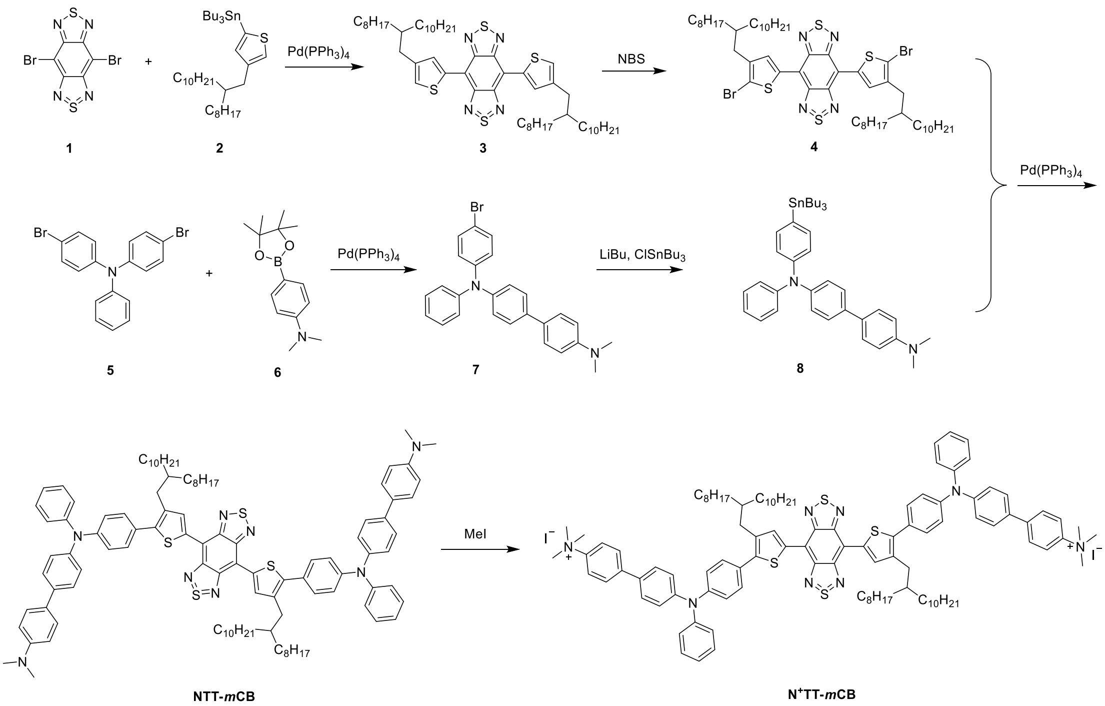

Figure S1. Synthesis route of N$^+$TT-*m*CB.

Synthetic route of **3**. Compound **1** (0.1 g, 0.28 mmol), **2** (0.39 g, 0.6 mmol), $Pd(PPh_3)_4$ (20 mg, 0.017 mmol) and 20 mL of toluene were added to a two-necked flask. After cooling to room temperature, the solvent was removed by rotary evaporation. The crude product was purified by silica gel column (hexane) to obtain the product (yield: 65%). ^{1}H NMR (400 MHz, $CDCl_3$), δ (ppm): 8.83 (2H, S), 7.28 (2H, S), 2.73-2.71 (4H, d, J = 8 Hz), 1.77 (2H, m), 1.36 (64H, m), 0.87 (12H, m).

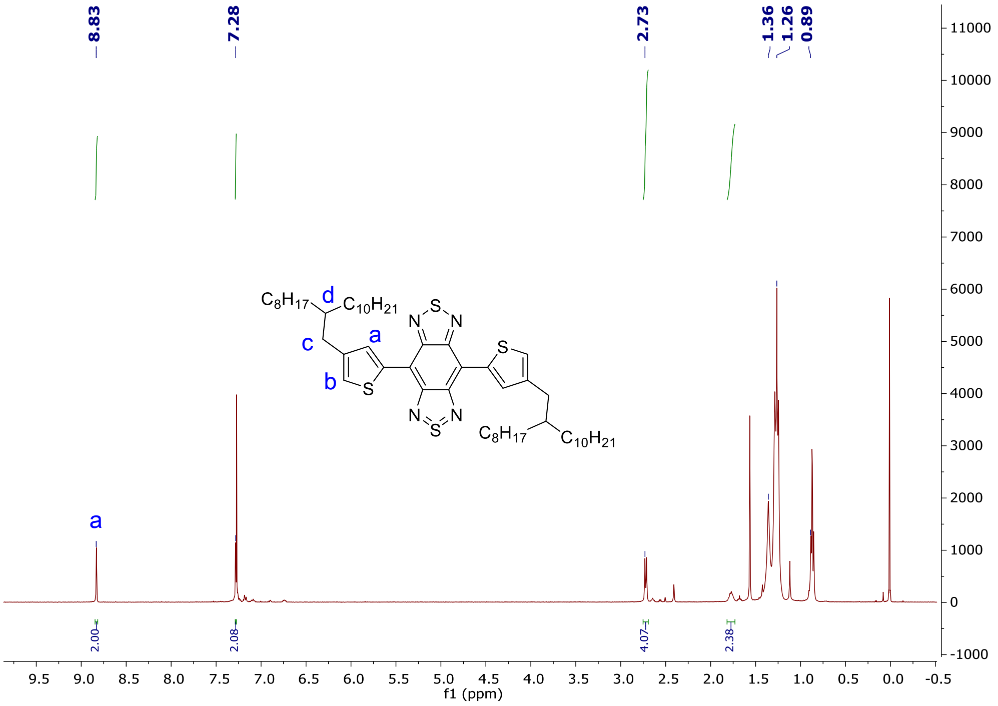

Figure S2. ^{1}H NMR spectrum of **3** ($CDCl_3$).

Synthetic route of **4**. Compound **3** (0.3 g, 0.33 mmol) was dissolved in a mixture of 10 mL of $CHCl_3$ and 10 mL of acetic acid under an argon atmosphere, and NBS (117 mg, 6.6 mmol) was added slowly over the course of 30 min in a mixture of 5 mL of $CHCl_3$ and 5 mL acetic acid at room temperature under the exclusion of light. The mixture was stirred overnight and then dried by condensed air. The crude product was purified by silica gel column to obtain the product (yield: 90%). ^{1}H NMR (400 MHz, $CDCl_3$), δ (ppm): 7.58-7.3 (8H, m), 7.29-7.25 (2H, m), 7.13-6.98 (5H, d, J = 8 Hz), 6.83 (2H, m).

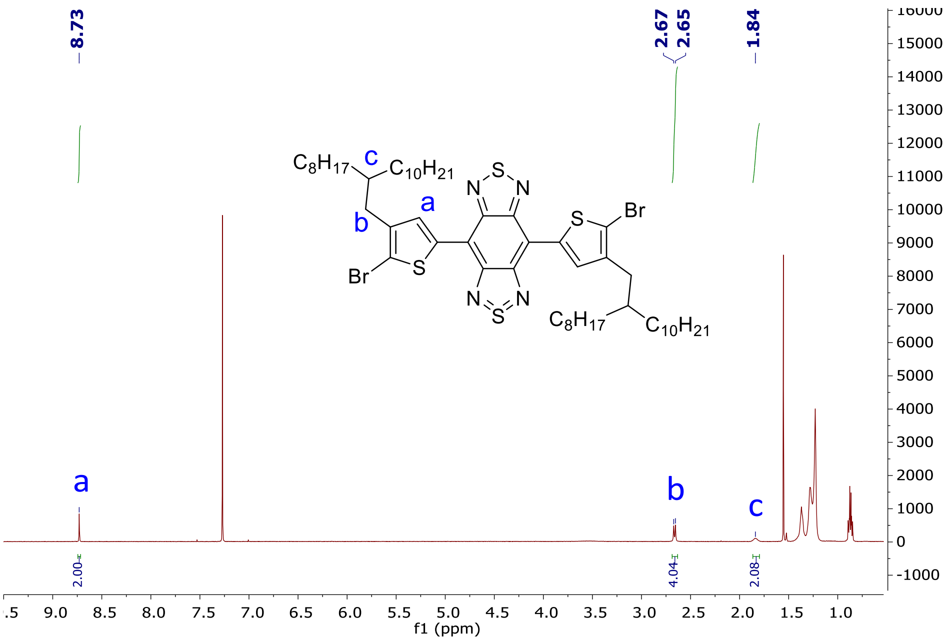

Figure S3. ^{1}H NMR spectrum of **4** ($CDCl_3$).

Synthetic route of **7**. Compound **5** (0.16 g, 0.4 mmol), compound 6 (0.15 g, 0.4 mmol), Pd $(PPh_3)_4$ (23 mg, 0.02 mmol) and K_2CO_3 (0.44 g, 3.2 mmol) were added to a 100 mL round-bottle flask. The flask was vacuumed and purged with dry nitrogen three times, and then 10 mL of THF and 2 mL of H_2O were added. The mixture was stirred at 80 °C overnight under N_2. After cooling to room temperature, the mixture was poured into water and extracted with DCM. The organic phase was combined and dried over Na_2SO_4. After solvent evaporation of the solvent, the residue was purified by column chromatography on silica gel (45% yield). ^{1}H NMR (400 MHz, $CDCl_3$), δ (ppm): 8.73 (2H, S), 2.67-2.65 (4H, d, J = 8 Hz), 1.84 (2H, m), 1.36 (64H, m), 0.87 (12H, m).

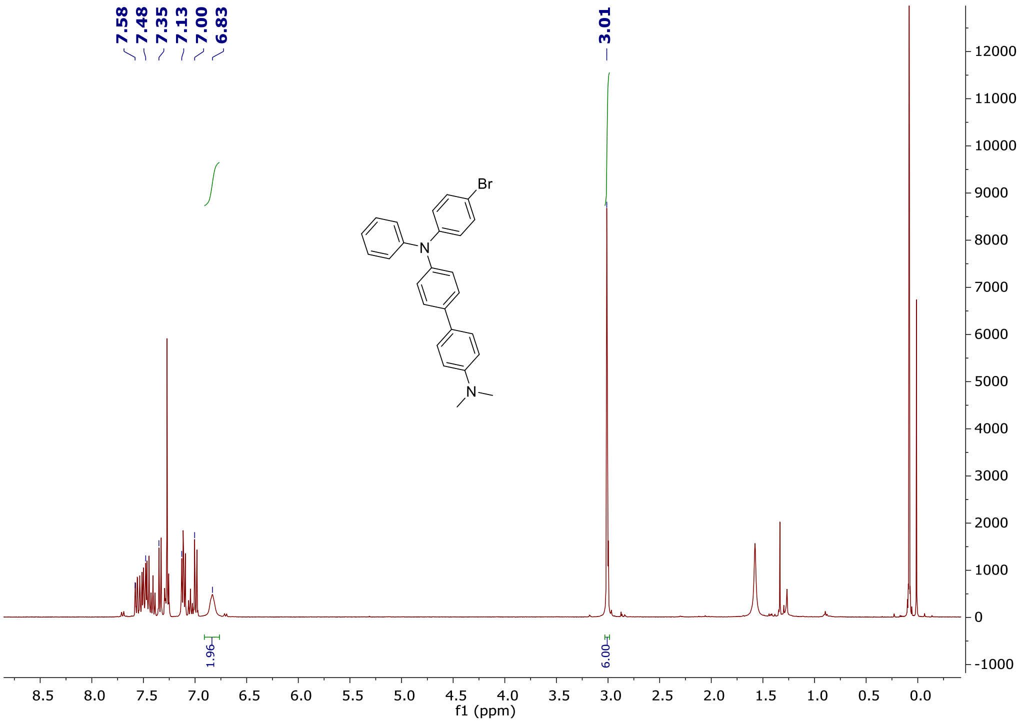

Figure S4. ^{1}H NMR spectrum of **7** ($CDCl_3$).

Synthetic route of **8**. Add nBuLi (0.7 mL, 1.7 mmol, 2.4 M in hexane) dropwise to a solution of **7** (1 g, 1.7 mmol) in THF (30 mL) at -78 °C. The reaction mixture was stirred for 1 h at -78 °C. Then, trimethyltin chloride (1.7 mL, 1.7 mmol, 1.0 M in THF) was added to the reaction at one portion. After stirring the mixture for 12 h at room temperature, KF solution was added to quench the reaction. The mixture was extracted with hexane three times, and the combined organic phase was dried with Na_2SO_4. After removing the solvent, the product was used directly without further purification.

Synthetic route of **NTT-*m*CB.** To a solution of compounds **4** (161 mg, 0.15 mmol) and **8** (261 mg, 0.4 mmol) in toluene (10 mL) was added Pd $(PPh_3)_4$ (4 mg). The mixture was stirred for 12 h at 100 °C. After cooling to room temperature, the mixture was poured into water and extracted with DCM. The organic layer was washed with saturated KF and brine before being dried over $MgSO_4$. After evaporation of the solvent, the residue was purified by column chromatography on silica gel to afford the product (yield: 55%). ^{1}H NMR (400 MHz, $CDCl_3$), δ (ppm): 8.92 (2H, S), 7.51 (12H, m), 7.33-7.20 (18H, m), 6.85-6.81 (4H, m), 3.02 (4H, s), 2.80 (4H, m), 1.84 (2H, m), 1.36 (64H, m), 0.87 (12H, m).

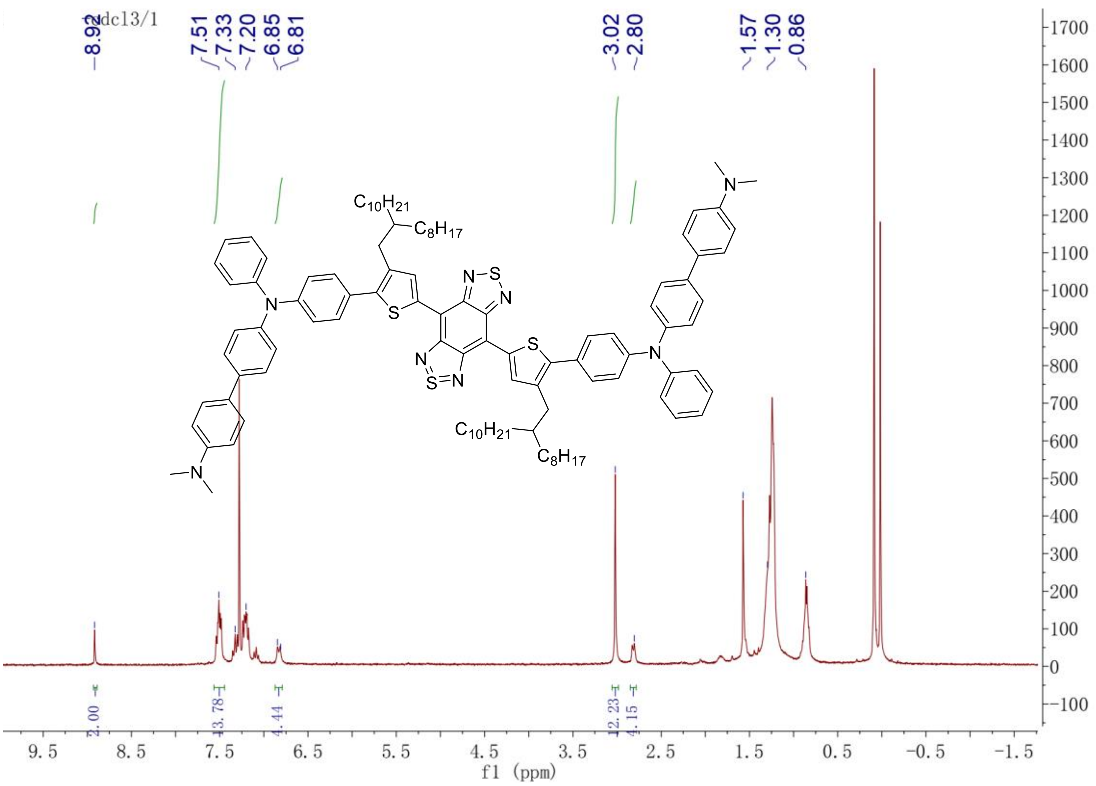

Figure S5. ^{1}H NMR spectrum of NTT-*m*CB ($CDCl_3$).

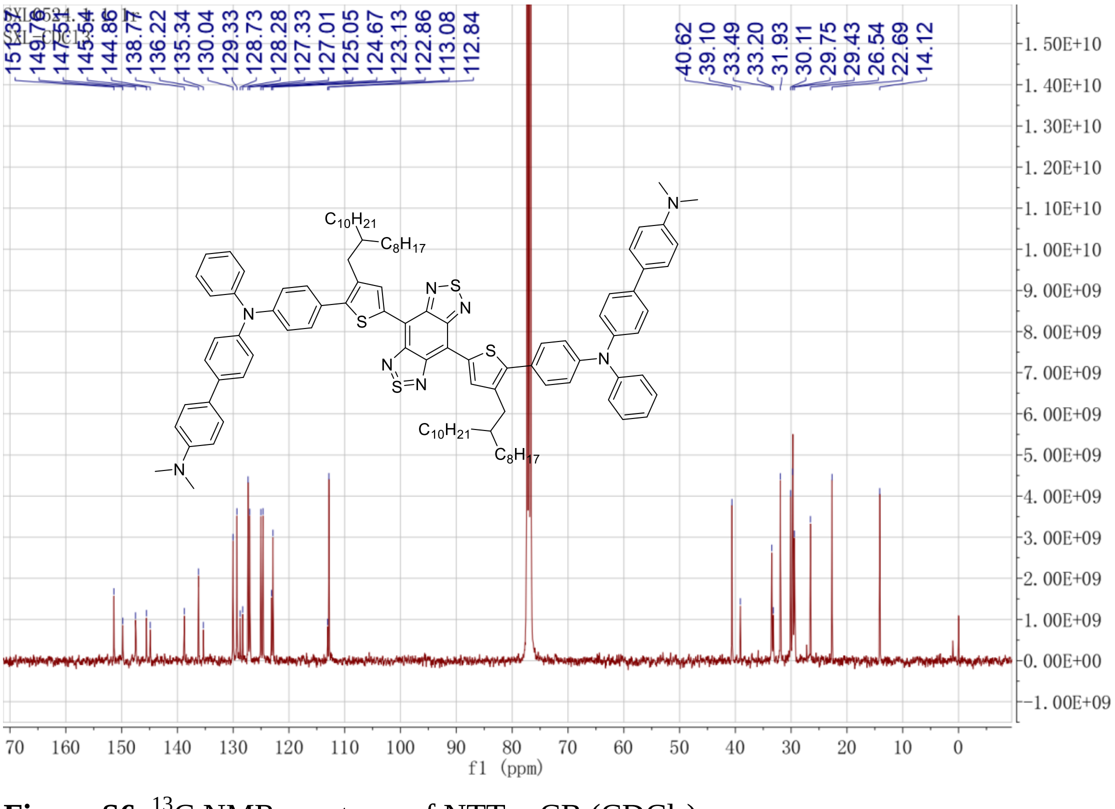

Figure S6. ^{13}C NMR spectrum of NTT-*m*CB ($CDCl_3$).

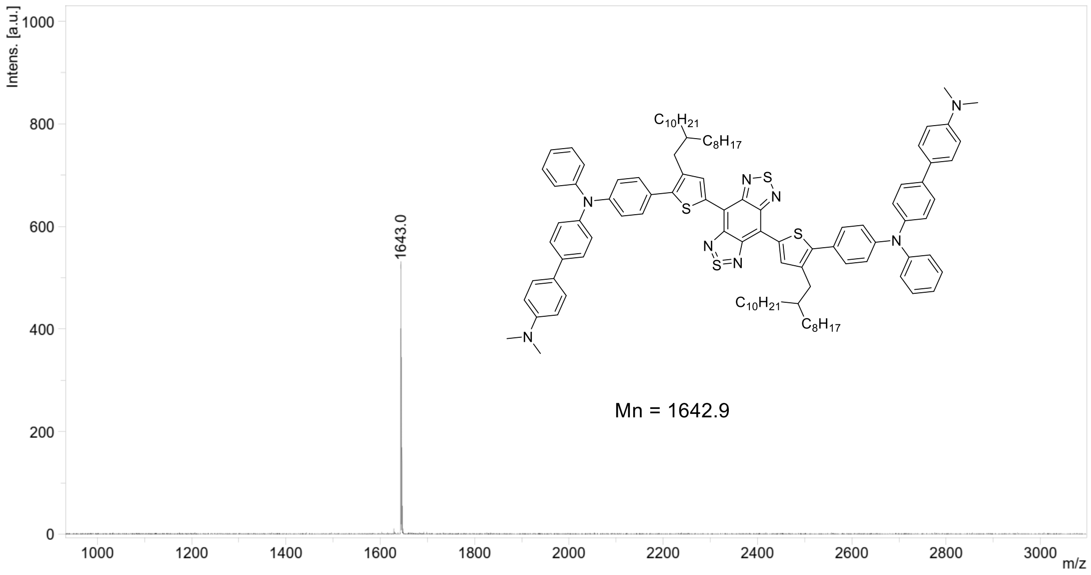

Figure S7. MALDI-TOF-MS spectrum of NTT-*m*CB.

Synthetic route of **N$^+$TT-*m*CB.**

To a solution of compound **NTT-*m*CB** (100 mg) in acetone (5 mL) was added MeI (1 mL). The mixture was refluxed overnight. After cooling to room temperature, the mixture was subjected to rotary evaporation. The crude product was purified by DCM washing several times.

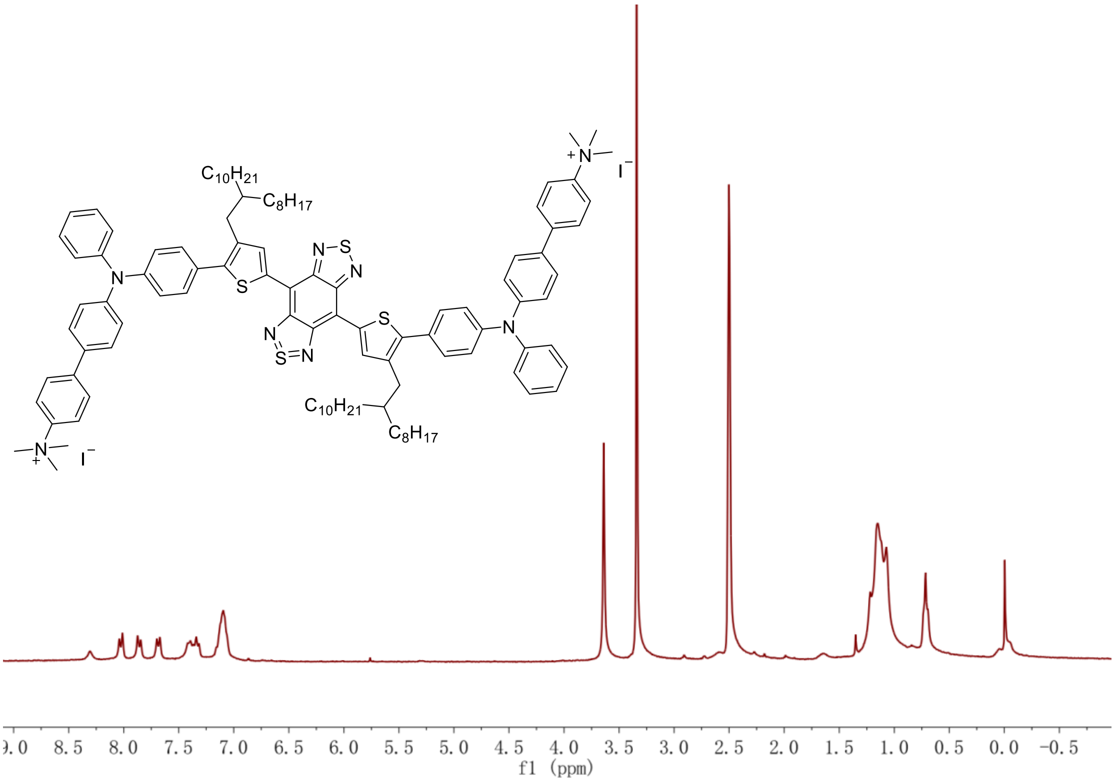

Figure S8. The ^{1}H NMR spectrum of **N$^+$TT-*m*CB** (DMSO-d6).

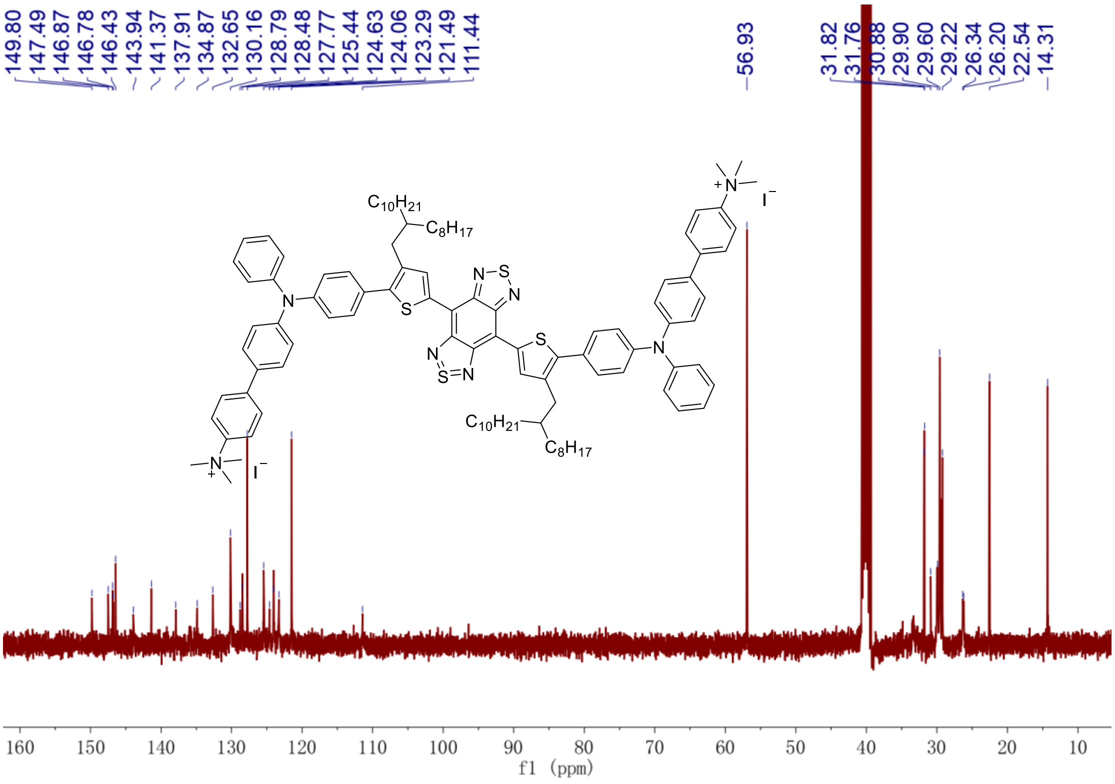

Figure S9. The ^{13}C NMR spectrum of **N^{+}TT-*m*CB** (DMSO-d6).

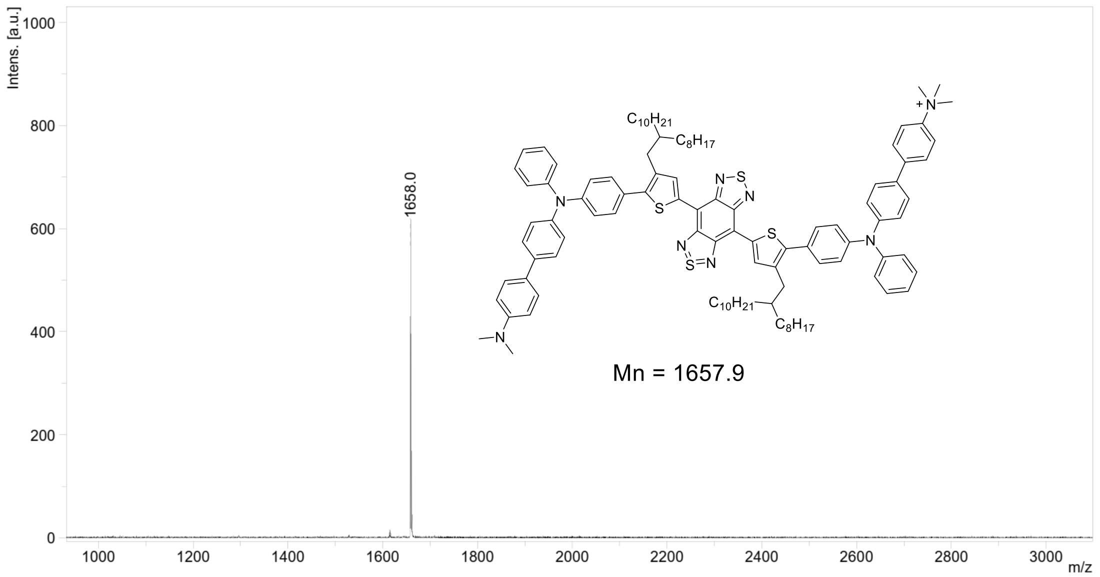

Figure S10. MALDI-TOF-MS spectrum of **N^{+}TT-*m*CB** (DMSO-d6).

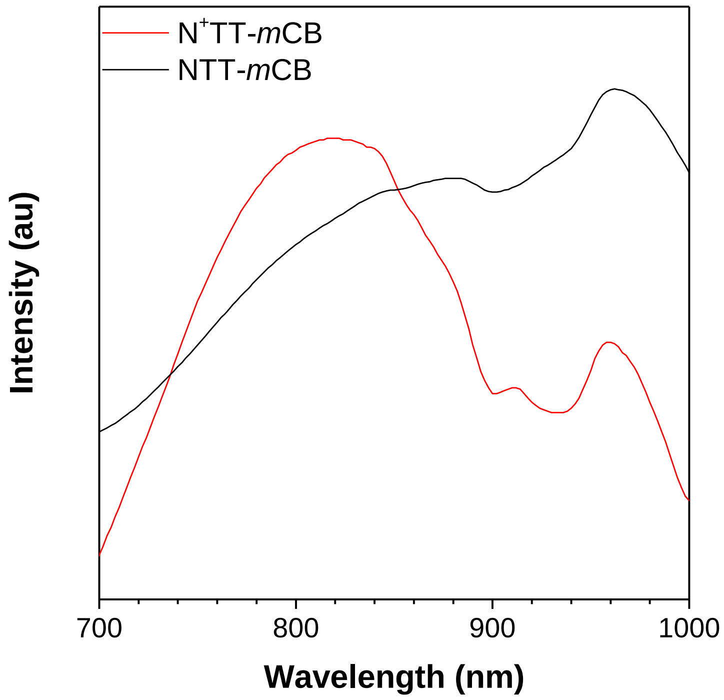

Figure S11. The normalized absorption spectrum of NTT-mCB NPs and N$^+$TT-mCB NPs.

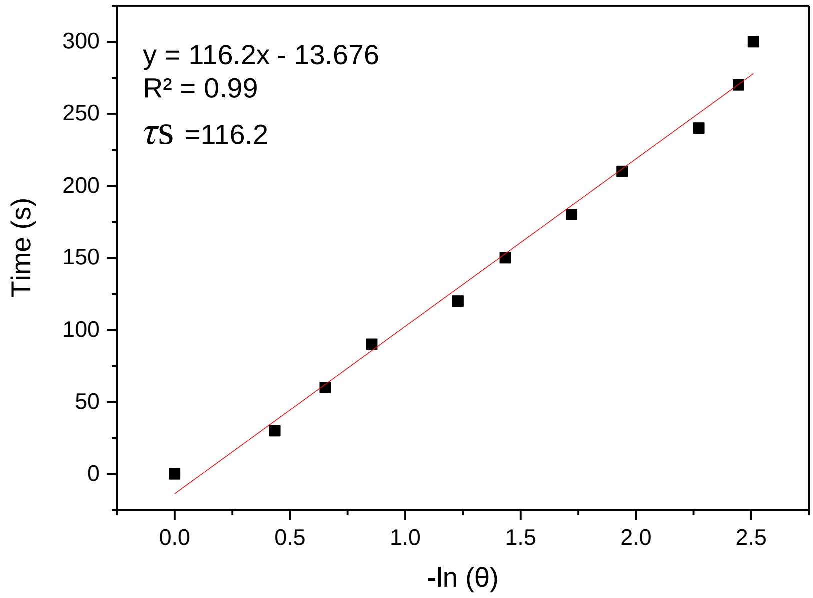

Figure S12. Plots of linear fitting time versus negative natural logarithm of driving force temperature.

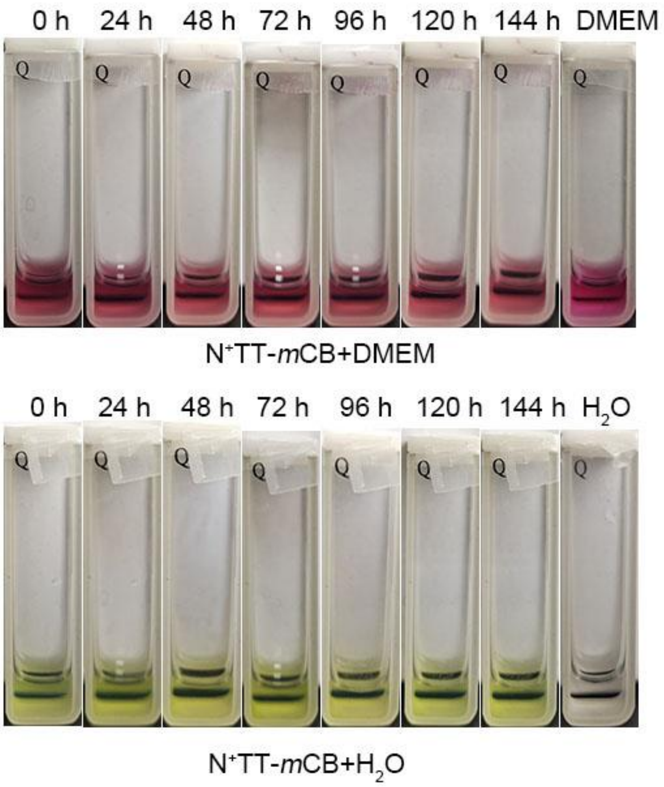

Figure S13. Images of N$^+$TT-*m*CB in DMEM and H_2O at room temperature from 0 h to 144 h.

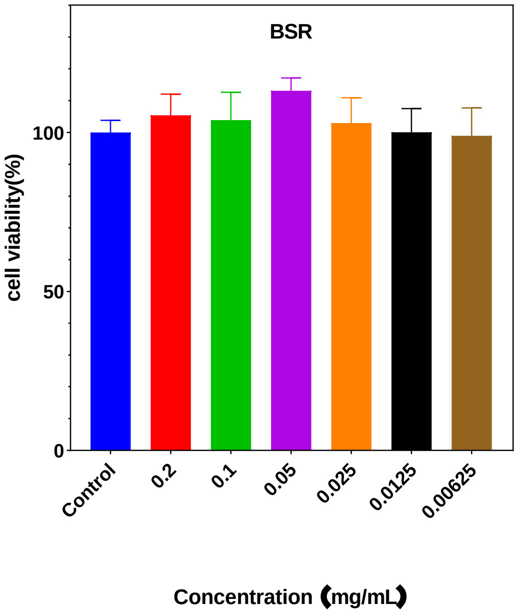

Figure S14. Cell vitality assay of BSR cells treated with N$^+$TT-*m*CB at 37 °C for 24 h. Bar graph represents means ± SD, n = 6. Statistical analysis of grouped comparisons was carried out by Student's *t* test.

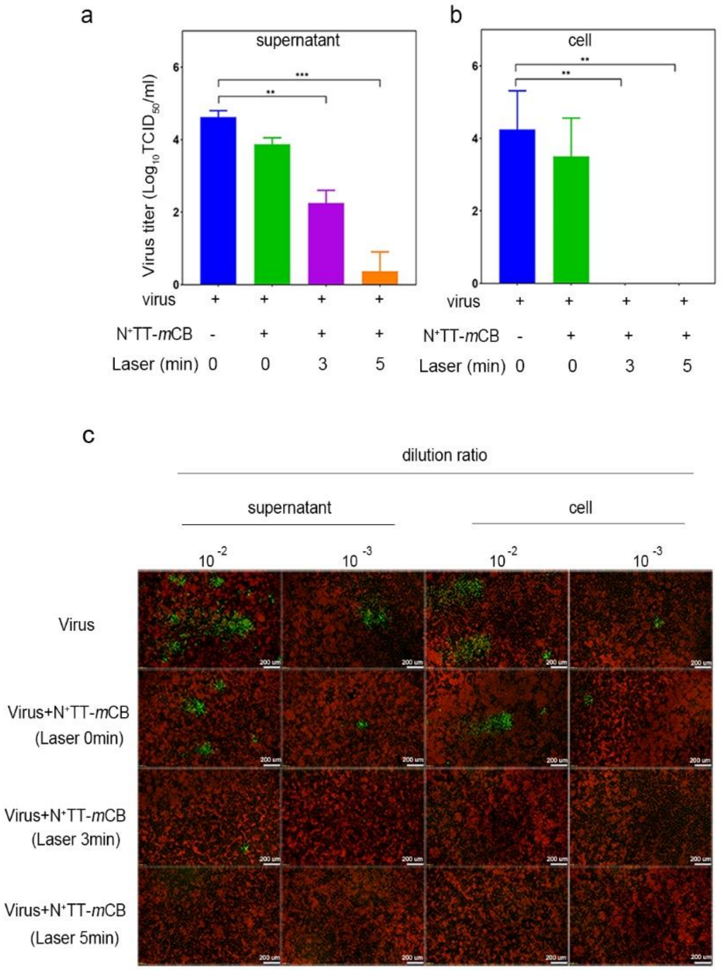

Figure S15. Antiviral effect of N$^+$TT-*m*CB in vitro. RABV titer in supernatant (**a**) and cells (**b**) with different treatments. (**c**) Representative fluorescence images of RABV in the supernatant and cells with different dilution ratios. Green represents virus-infected cells, and red represents cells without virus infection. The cells were strained with FITC anti-Rabies monoclonal globulin (1:200) and Evans blue (1:500). Bar graph represents means ± SD, n = 3. Statistical analysis of grouped comparisons was carried out by Student's *t* test (**P < 0.01, ***P < 0.001).

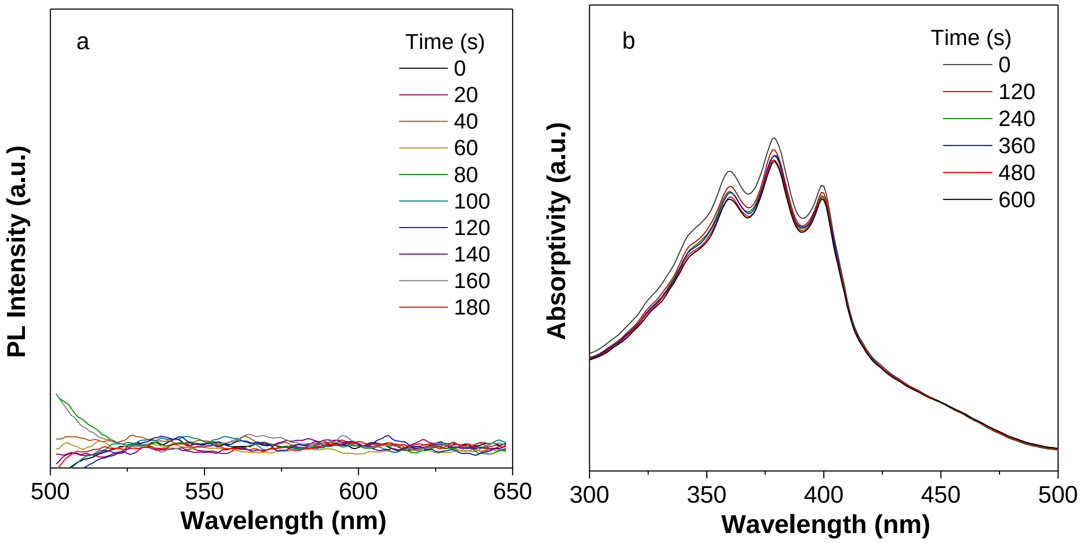

Figure S16. The PL spectrum of HPF (**a**) and UV spectrum of ABDA (**b**) in the presence of N^+TT-*m*CB nanoparticle under 808 nm laser irradiation.

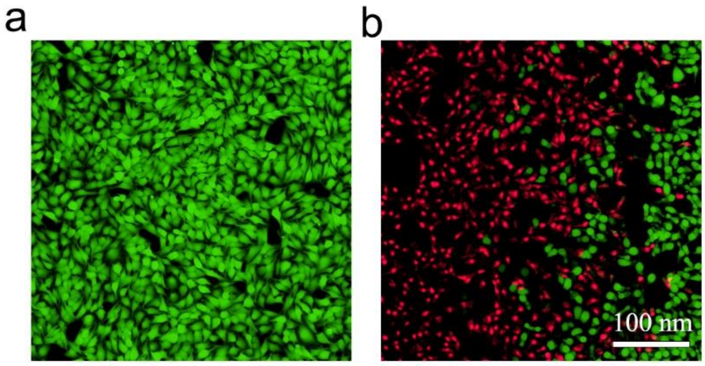

Figure S17. Live/dead cell staining. Normal cells without any treatment (**a**) and cells were treated with N^+TT-*m*CB (0.2 mg/mL) for 5 min 808 nm laser irradiation (0.45 W/cm^2) (**b**). The cells were strained with Propdium iodide and Calcein AM. Green represents live cells and red represents dead cells.

Table S1. Blood and biochemical indexes of three groups of mice (PBS, N^+TT-*m*CB with one irradiation and N^+TT-*m*CB with two irradiation, n = 3. Major items: RBC: red blood cell, MCV: mean corpuscular volume, RDWa: remote desktop web access, HCT: haematocrit, PLT: platelets, MPV: mean platelet volume, WBC: white blood cell, HGB: haemoglobin, MCH: mean corpuscular haemoglobin, MCHC: mean corpuscular haemoglobin concentration, LYM: lymphocyte, MONO: monocyte, ALB: albumin, TP: total protein, GLB: globulin, ALP: alkaline phosphatase, CREA: creatinine, BUN: blood urea nitrogen, GLU: glucose)

Blood Indexes

Test	Control	N^+TT-*m*CB (**1** Laser)	N^+TT-*m*CB (**2** Laser)	Normal Range
RBC (10^9/L)	9.125±0.255	8.81±0.32	8.24±0.79	6.50-10.10
MCV (fL)	55.2±4.6	56.45±4.65	54.23±4.15	42.3-55.9
RDW%	20.75±2.25	20.7±1.7	16.16±3.1	0.0-99.9
RDWa (fL)	32.6±0.7	34.2±1.5	33.9±9.79	0.0-99.9
HCT%	50.05±3.05	48.15±3.75	44.5±1.57	32.8-48.0
PLT (10^9/L)	1624±19	1701.5±136.5	785±115.65	250-1540
MPV (fL)	5.45±0.15	5.35±0.25	6.33±0.35	0.0-99.9
WBC (10^9/L)	8.1±1.3	7.2±0.8	11.46±0.92	2.67-13
HGB (g/dL)	15.95±0.25	15.45±0.85	14.2±1.05	10.0-16.1
MCH (pg)	17.65±0.45	17.55±0.35	17.26±0.47	13.7-18.1
MCHC (g/L)	31.8±2.5	30.35±2.05	31.9±1.56	29.5-35.1
LYM (10^9/L)	5.55±0.95	4.3±0.6	8.64±0.75	1.3-8.4
MONO (10^9/L)	0.55±0.15	0.65±0.05	0.25±0.14	0.0-0.3
LYM%	72.85±5.45	63.3±5	75.4±4.12	0.0-99.9

Biochemical Indexes

Test	Control	N^+TT-*m*CB (**1** Laser)	N^+TT-*m*CB (**2** Laser)	Normal Rangers
ALB (g/L)	33.8±0.6	31.5±0.2	28.67±2.51	25.0-48.0
TP (g/L)	68±1.2	65.25±1.85	57.85±1.06	36.0-66.0
GLB (g/L)	34.1±0.6	33.75±2.05	37.57±11.98	\
A/G	0.99±0	0.935±0.065	0.79±0.16	\
ALT/GPT	58.5±5.5	58±11	64±30.5	28-132
ALP (U/L)	138±11	173±6	117.67±23.86	62-209
CREA (umol/L)	<10.0	<10.0	13.45±3.75	18.0-71.0
BUN (mmol/L)	8.145±0.805	8.945±0.315	5.47±0.66	6.40-10.40
BUN/Cr	\	\	454.16±155.2	\
GLU (mmol/L)	8.335±0.235	7.81±0.86	7±0.32	5.00-10.67

Table S2. The expression of inflammatory factors in tissues was detected by relative quantitative real-time PCR. The primers (TNF-α, IL-1β, IL-6, IL-10 and IFN-β) used for qPCR are as follows.

Target Gene	Direction	Sequence (5'-3')
TNF-α	Forward	GGACTAGCCAGGAGGGAGAACAG
	Reverse	GCCAGTGAGTGAAAGGGACAGAAC
IL-1β	Forward	CACTACAGGCTCCGAGATGAACAAC
	Reverse	TGTCGTTGCTTGGTTCTCCTTGTAC
IL-6	Forward	CTTCTTGGGACTGATGCTGGTGAC
	Reverse	TCTGTTGGGAGTGGTATCCTCTGTG
IL-10	Forward	AGAGAAGCATGGCCCAGAAATCAAG
	Reverse	CTTCACCTGCTCCACTGCCTTG
IFN-β	Forward	AAGAGTTACACTGCCTTTGCCATCC
	Reverse	CACTGTCTGCTGGTGGAGTTCATC
